# Correlation Networks Provide New Insights into the Architecture of Testicular Steroid Pathways in Pigs

**DOI:** 10.3390/genes12040551

**Published:** 2021-04-09

**Authors:** Annie Robic, Thomas Faraut, Katia Feve, Sarah Djebali, Armelle Prunier, Catherine Larzul, Laurence Liaubet

**Affiliations:** 1 GenPhySE, Université de Toulouse, INRAE, ENVT, 31326 Castanet Tolosan, France; thomas.faraut@inrae.fr (T.F.); katia.feve@inrae.fr (K.F.); sarah.djebali@inserm.fr (S.D.); catherine.larzul@inrae.fr (C.L.); laurence.liaubet@inrae.fr (L.L.); 2 Institut National de Recherche en Santé Digestive (IRSD), Université de Toulouse, INSERM, INRAE, ENVT, Université Paul Sabatier (UPS), 31024 Toulouse, France; 3 PEGASE, INRAE, Institut Agro, 35590 Saint Gilles, France; armelle.prunier@inrae.fr

**Keywords:** porcine testis, co-expression network, steroids synthesis, WGCNA, testosterone, estrogens, androstenone, *CYP11A1*, HSD17B3, AKR1C

## Abstract

Steroid metabolism is a fundamental process in the porcine testis to provide testosterone but also estrogens and androstenone, which are essential for the physiology of the boar. This study concerns boars at an early stage of puberty. Using a RT-qPCR approach, we showed that the transcriptional activities of several genes providing key enzymes involved in this metabolism (such as *CYP11A1*) are correlated. Surprisingly, *HSD17B3*, a key gene for testosterone production, was absent from this group. An additional weighted gene co-expression network analysis was performed on two large sets of mRNA-seq to identify co-expression modules. Of these modules, two containing either *CYP11A1* or *HSD17B3* were further analyzed. This comprehensive correlation meta-analysis identified a group of 85 genes with *CYP11A1* as hub gene, but did not allow the characterization of a robust correlation network around *HSD17B3*. As the CYP11A1-group includes most of the genes involved in steroid synthesis pathways (including *LHCGR* encoding for the LH receptor), it may control the synthesis of most of the testicular steroids. The independent expression of *HSD17B3* probably allows part of the production of testosterone to escape this control. This CYP11A1-group contained also *INSL3* and *AGT* genes encoding a peptide hormone and an angiotensin peptide precursor, respectively.

## 1. Introduction

The regulation of the synthesis of testicular steroids is of major importance in boars, not only for the control of reproduction but also for the specific problem of boar taint [1]. In mammals, production of sex steroids by Leydig cells is, at least in part, under the endocrine control of both gonadotropins (LH and FSH) and under the paracrine control of testosterone and estradiol [2]. The action of these hormones is mediated by their specific receptors (encoded by *LHCGR*, *FSHR*, *AR* and *ESR1/ESR2* genes, respectively). In mammals, the production of LH and FSH by pituitary glands is under the control of GnRH (gene *GNRH1*) produced by hypothalamus. In contrast to other mammals; however, the porcine genome contains a second gene *GNRH2* producing a second gonadotropin. Its expression is ubiquitous but its highest expression in testes suggests an important role in testicular function [3]. GnRH2 and its receptor GnRHR2 may control testicular testosterone synthesis independently of LH [4]. A second specific feature of testicular steroid production in boars is the production of high amounts of unconjugated and conjugated estrogens as well as of 16-unsaturated steroids (androstenone) [5]. In contrast to human testes, which are only able to produce estrogens in Sertoli cells, the porcine testes produce estrogens in Leydig cells [6,7]. Again, in contrast to humans where testes produce only about 20% of circulating estrogens, the remainder being produced by aromatization of testosterone in peripheral tissues [8], boar testes seem to be the major source of estrogens [5]. A third specific feature of boar testes is the high production of androstenone (5α-androst-16-en-3-one) derived from pregnenolone. Androstenone is released in blood and can be exported in saliva where it plays a pheromone role for attracting females in estrus. Due to its lipophilic properties, androstenone can accumulate in adipose tissue, causing boar taint when fat is heated [1,5]. Once again, in contrast to humans, boar testes produce large amounts of 5α-reduced steroids (epiandrosterone, androstanediol, and androstenone). Nevertheless, the 5α reduction of testosterone to dihydrotestosterone (DHT) has never been described in porcine testes [9]. With these numerous features relative to endocrine functions, it is not surprising that boars differ from other mammals in terms of testicular morphology: The volume percentage of Leydig cells per whole testis of mature boars is very high [10].

Genes coding for most of the important proteins involved in the testicular biosynthesis of steroids are known [11,12] and have been described in pigs [5]. The biosynthesis of sex steroids could be described by an organization around two intermediate compounds and four classes of steroids (estrogens, testosterone, androstenone and other androgens) (see Appendix A). The enzyme encoded by *CYP11A1* catalyzes three sequential reactions from cholesterol into pregnenolone. In all mammals, the transformation of pregnenolone in androstenedione opens the common pathway to estrogens and androgens [5,12]. Besides, in pigs, the transformation of pregnenolone in androstenone is also possible. In pigs, a unique gene *HSD3B1* encodes for the hydroxy-delta-5-steroid dehydrogenase and *CYP17A1* encodes for the cytochrome P450 17α-hydroxylase/17,20-lyase, these two enzymes being essential [5]. Genes from the cluster of *CYP19A* (in pigs three genes are suspected, [5,13]) encode the aromatase enzyme essential for the transformation of androstenedione in estrogens. The transformation of androstenedione in testosterone needs the expression of a gene coding for a 17β-hydroxysteroid dehydrogenase [11]. Usually, the gene *HSD17B3* is considered to encode this enzyme [11,14], but the *AKR1C3* gene would also be involved in humans or *HSD17B1* in mice [15,16]. In pigs, former investigations showed an intense activity of *HSD17B3* in testes but not in ovaries [17]. In addition, *HSD17B1* appeared inactive in testis, unlike at least two genes from AKR1C cluster/family [17]. A previous study in pigs [18] showed that the transcriptional activities of *CYP11A1*, *CYP17A1, CYP19A* and *AKR1C4* (the name of porcine and human AKR1C genes are independent) were significantly correlated in testes. Surprisingly, *HSD17B3*, known to be a key gene for the production of testosterone, was excluded from this group [18].

The main objective of this study was to improve the knowledge on genes involved in the steroid synthesis in porcine testes and to understand the genetic architecture of testicular steroid pathways in pigs. We proposed to study the testicular transcriptome from whole testis fragments collected on animals that were not sexually mature. These animals are not in the same stage of pubertal development and the balance between the different cell types (i.e., Leydig and Sertoli cells) may differ between the testes collected. We propose to deal with this heterogeneity by analyzing a large number of animals and by an approach based on the characterization of common relationships between gene expressions. We propose a first analysis focused on a list of genes known to be involved in steroid metabolism by using data from RT-qPCR. A whole-genome correlation analysis (non-targeted mRNAseq) was conducted next in order to expand communities of genes around *CYP11A1* and *HSD17B3* genes.

## 2. Materials and Methods

### 2.1. Animals, Sample Collection

A large population of intact males was produced by the three French breeding companies of the Alliance R&D group (Axiom, Choice Genetics, Nucléus, IFIP) involved in the UtOpIGe project (ANR-10-GENOM_BTV-015). This population was balanced between purebred Pietrain and crossbred Pietrain × Large White [19]. Animal husbandry, sampling and slaughtering procedures were previously described [10]. Animals were slaughtered near 160 days (±3 weeks) of age, namely during pubertal development. Pigs from European breeds (Pietrain, Large White) are considered sexually mature (testes able to produce sperm and hormones) at eight months. All animals were evaluated for testosterone and 17β-estradiol levels, a week before slaughter (exceptionally 2 weeks). Blood samples were collected in the jugular vein, immediately centrifuged at 2500× *g* for 10 min at 4 °C, and the plasma was stored at −20 °C until analysis. The detection limits were 0.28 ng/mL and 7 pg/mL for testosterone and 17β-estradiol, respectively.

Samples of testes (several pieces with a total weight of 3–4 g) were collected within 60 min after the death of the animal and immediately frozen in liquid nitrogen. The total amount of samples was ground to a fine powder during 1 min in a liquid nitrogen–cooled grinder with stainless steel beads. Total RNA was extracted with TRIzol reagent (Invitrogen) and all samples were subjected to an on-column-purification step with the NucleoSpin RNA II kit (Macherey-Nagel) including a DNase digestion to remove contaminating genomic DNA. RNA Quality was evaluated by a ND-1000 Spectrophotometer (NanoDrop Technologies, Wilmington, DE, USA) and an Agilent Bioanalyser 2100 (Agilent Technologies, Santa Clara, CA, USA). The RNA integrity numbers were higher than 8.9 for all RNAs retained. In total, RNA was extracted in 80 testicular samples [20]. For RT-qPCR, we analyzed a large number of samples with a balanced genetic origin (21 Pietrain and 21 Pietrain × Large White). For mRNA-seq, we chose to consider two distinct batches including a large number of samples of Pietrain and Pietrain × Large White, respectively. Breed and plasma concentrations of testosterone and 17β-estradiol are described in Supplementary Table S1.

### 2.2. Gene Expression Analysis by RNA-Seq

After a polyA+ selection, the Illumina TruSeq mRNA kit was used to generate libraries for stranded paired-end sequencing. These libraries were prepared in two batches. The first batch included testes from 18 Pietrain animals (‘Seq-P’), and 15 months later, the second batch included testes from 19 crossed animals (‘Seq-PLW’). Even if the 37 mRNA-seq were obtained in a single Novaseq sequencing run, we suspect the existence of a batch effect between mRNAseq-P and mRNAseq-PLW data.

The reads of total-RNA-seq from six pubertal testes deposited in the NCBI under accession number PRJNA506525 (NCBI-SRA BioProject) were retained (3 Pietrain and 3 crossed animals from the same population). For a preliminary analysis, three total-RNAseq datasets produced by EMBL from 2-year-old boar testes (EMBL-pig4/ERR3417952, EMBL-pig5/ERR3417904, EMBL-pig6/ERR3418012) were considered.

Reads were mapped to the porcine genome by STAR with “pair ends” option [21] and further processed by RSEM (version 1.3.0) [22] to obtain gene and transcript expressions. The genome assembly (Sscrofa11.1) used for all alignments corresponded to GenBank Assembly ID GCA_000003025.6, and *Ensembl* gene annotation v-90 was used [23]. RSEM calculates fragments per kilobase per million and transcripts per million (TPM) values. In this study we use log_2_(TPM+0.01) to obtain normalized data (Supplementary Figure S1A,B).

### 2.3. Gene Expression Analysis by RTqPCR

The subset for RT-qPCR evaluation (‘qPCR’) included 21 Pietrain and 21 crossbred (Pietrain × Large White) animals [20].

Reverse-transcription was performed with the high-capacity cDNA reverse transcription kit (Applied Biosystems #4368814) using 2 μg of total RNA from each sample as a template and dN_6_ random primers, according to the manufacturer’s instructions.

We designed primers to evaluate the level of 33 transcripts. This list contained several transcripts known to be involved in steroid synthesis already analyzed in RT-qPCR [18], as *HSD17B3*, *CYP11A1* or *CYP17A1,* or not, as *STAR* and *HSD3B1*. We added some other genes, which have a priori no important role in testicular steroids metabolism in testis as *HSD11B1* and *HSD11B2* [9]. Additional genes were included, namely *GSTA2*, *STARD6* and *FMO1* in [24] and *DHRS4* and *SULT2A1* [25,26]. Finally, we added a few genes with a known expression in testis. Sequences of all primers are given in Supplementary Table S2.

We evaluated the quantity of transcripts using the real-time polymerase chain reaction (PCR) technology on reverse transcription products from each gene with a protocol already detailed [18]. All measurements were performed on the same plate, and no reference sample was used. Four dilution points containing a mix of complementary DNA were used for each gene and each tissue to determine PCR efficiency (*E*). Since efficiency levels were similar for all the genes measured (including the reference genes), results were expressed as 2^(Ct_ref − Ct_gene)^ × 1000 in arbitrary units. Two reference genes (*LAPTM4A* and *SUGP1*) were used for RT-qPCR normalization. These two genes were selected among genes with very low variation in mRNAseq expression levels and genes not known to produce circRNAs [20].

### 2.4. Expression Quantification of Genes Localized in Genomic Clusters (CYP19A, AKR1C and SULT1C)

Three *CYP19A* genes are characterized in pigs by their distinct transcripts which differ by few nucleotides [13]. A cluster of three genes is expected but the reference genome assembly Sscrofa11.1. and associated *Ensembl* annotation refers to only one gene as *CYP19A1* (with a main transcript NM_214430, i.e., *CYP19A2* [23]). The RSEM quantification therefore only evaluates the expression of all transcripts from the three *CYP19A* genes. To perform a reliable quantification by RT-qPCR, we took into account the high similarities between *CYP19A1, CYP19A2*, and *CYP19A3* to propose only one evaluation for these three transcripts from the three *CYP19A* genes.

Four AKR1C genes are distinguishable in pigs by their respective transcripts [5]. According to what is observed in humans, a cluster of four AKR1C genes associated with an *AKR1E2* gene is expected in the porcine genome. Porcine AKR1C genes are not orthologous to human AKR1C and an identical name does not mean that this gene is involved in the same reactions [5] (details in Supplementary Table S2). To perform a reliable quantification by RT-qPCR, we took into account the high similarities between *AKR1C1* and *AKR1C2* (*AKR1C-pig1* and *AKR1C-pig3* [5,18]) to propose only one evaluation for these two *AKR1C* transcripts. The reference genome assembly Sscrofa11.1 contains only one gene referenced by *Ensembl* as *AKR1C2* but a blast demonstrates that it is *AKR1C4* (*AKR1C-pig6* [5,18]). Here again the RSEM quantification only evaluates the quantity of all transcripts from the four *AKR1C* genes. Nevertheless, these transcripts could be involved in distinct pathways.

In the human and mouse genomes, a cluster of two or three genes SULT1C and two pseudogenes are found. The reference genome assembly Sscrofa11.1 contains one large gene (ENSSSCG00000028691) and two genes localized (ENSSSCG00000022153 and ENSSSCG00000037616) in one of its introns. RSEM counts appeared focused on ENSSSCG00000028691 in RNA-seq considered here.

### 2.5. Statistical Analyses

A part of correlations analyses were performed with the R software [27]. Coefficients of correlation were calculated between variables with the “psych” library. Another part of correlations analyses were performed with MS-Excel (Part of Microsoft-office, version 2016-pro+,Microsoft Corporation, Redmond, WA, USA) [28]. Correlations were calculated using the Pearson’s coefficient. Correlation analyses using the weighted correlation network analysis package, WGCNA 1.69 [29], were performed with the R software (version 4.0.3) [27]. A filtered dataset of 17,170 genes was used to keep genes with a median expression arbitrary chosen above -6. The analysis was conducted following the WGCNA developers’ recommendations [30]. The following parameters were used: softPower 10 and 9 for mRNAseq-PLW and mRNAseq-P dataset respectively, and a minimum module size of 50.

The hierarchical clustering analyses (HCA) were performed on the Galaxy platform proposed by Sigenae [31]. This tool is part of a set of statistical tools made available by members of the BIOS4BIOL group. All clusters were done with the “ward” agglomeration method as suggested by developers [32]. Principal component analysis (PCA) was performed with the “FactoMineR” library with the R software [27].

### 2.6. Functional Enrichment Analyses

Enrichment analysis of correlated groups of genes was conducted using the Genecodis4 software [33] with the following parameters: minimum three genes for enriched annotation, co-annotation using biological process gene ontology and KEGG pathways databases. Results are considered as significant if the *p*-value (*p*) of the false discovery rate (FDR) test of Benjamini and Hochberg [34] is <0.05.

The identification of possible regulations by transcription factors was realized using an enrichment analysis with the Genecodis4 software using the DoRothEA Regulons database [35].

## 3. Results

### 3.1. RT-qPCR-Correlation Analyses

**Figure 1:**
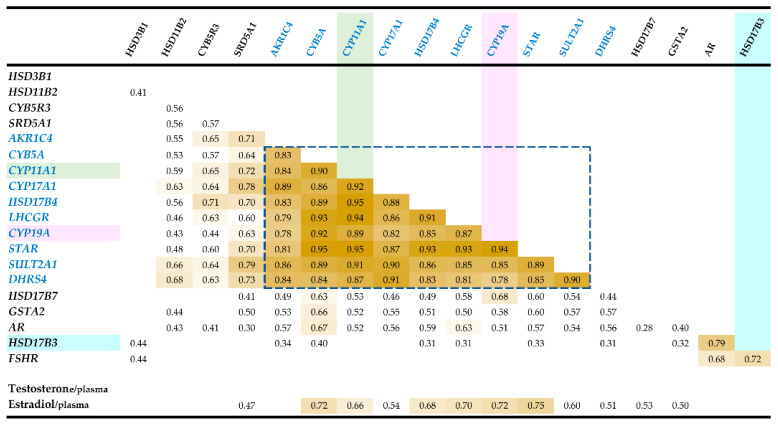
Correlations detected by the analysis of RT-qPCR results. These RT-qPCRs were performed on 33 genes, but only correlations concerning 18 genes and with *p* < 0.01 are reported. The correlations between the testicular expression of genes and plasma concentrations of testosterone and 17β-estradiol are presented at the bottom of this diagram.

Results were obtained for the 42 testicular samples (subset ‘qPCR’ and list in Add-Table S1) and transcriptional relative abundance of the 33 transcripts (list in Supplementary Table S2) was characterized. Ten genes (*CYP11A1, CYP17A1, CYP19A, AKR1C4, HSD17B4, LHCGR, CYB5A, STAR, SULT2A1,* and *DHRS4*) are highly correlated (0.79 < *r* < 0.95, *p* < 0.00001). *AR* appeared significantly correlated with *HSD17B3* and *FSHR* (Figure 1). Most of the other correlations which were not reported in Figure 1 are very weak, except for a strong correlation between *INHA* and *HSD3B1* (r = 0.74).

The correlations between the expression of genes and plasma concentrations of testosterone and 17β-estradiol were estimated (Figure 1). Plasma 17β-estradiol is significantly correlated with the expression of 12 genes (*p* < 0.01). In contrast, no significant correlation is observed between plasma testosterone and the 33 genes tested here (*p* < 0.01).

### 3.2. Detection of Correlation Groups in mRNAseq

#### 3.2.1. Hierarchical Clustering mRNAseq

**Figure 2:**
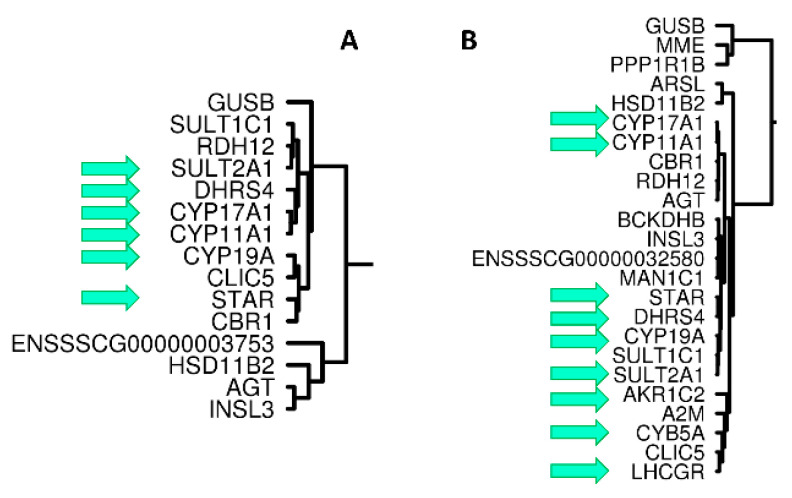
Part of HCA of mRNAseq-P (**A**) and from mRNAseq-PLW (**B**). Among more than 9000 genes, an HCA was built by retaining the top 100 genes with the highest variance (see Supplementary Figure S2). We selected for this figure the part of these HCA including some genes already identified as highly correlated in this study. A green arrow indicated the genes present in the group of genes correlated to *CYP11A1* and characterized by RT-qPCR (see Figure 1).

Two distinct batches of mRNA-seq, mRNAseq-P (18 Pietrains) and mRNAseq-PLW (19 crossbred animals) were analyzed. Two hierarchical cluster analysis (HCA) were performed on the expression of 9395 genes with the highest transcriptional activity. The top 100 genes with the largest variance were retained to perform the hierarchical clustering using Pearson’s correlations as distance (agglomeration with Ward). On the HCA performed on mRNAseq-P data, the group of genes clustered with the strongest correlation (see Supplementary Figure S2A) contains *SULT2A1*, *CYP11A1*, *CYP17A1* and *DHRS4* as well as *SULT1C1* and *RDH12* (Figure 2A). On the HCA performed on mRNAseq-PLW data, a similar group of genes clustered with the strongest correlations is observed. It contains *SULT2A1*, *CYP11A1*, *CYP17A1*, *DHRS4*, and also *STAR*, *CYP19A* as well as eight other genes (Figure 2B, Supplementary Figure S2B).

#### 3.2.2. Identification of Correlation Modules Containing CYP11A1 and HSD17B3

A without a priori approach was used to identify groups of correlated genes from the two transcriptomic datasets. Our purpose was to identify groups of genes linked by weak correlation distance (i.e., identify networks of co-expressed genes) and containing *CYP11A1* and other genes involved in steroid synthesis. A weighted correlation network analysis (WGCNA) [29] was performed on the two datasets of mRNAseq after filtration, and 17,170 genes were analyzed. First of all, PCA analysis confirms that these two datasets of mRNA-seq should be analyzed separately (Supplementary Figure S1C). Besides, based on the correlation among samples, a clustering dendrogram was drawn to verify that both datasets did not contain any outliers (Supplementary Figure S1D).

In the mRNAseq-PLW dataset, 67 modules of genes were identified: *HSD17B3* and *CYP11A1* were identified in two modules of 335 and 509 genes, respectively. The WGCNA analysis of the mRNAseq-P dataset identified 65 modules. *HSD17B3* and *CYP11A1* were identified in two modules of 585 and 729 genes, respectively. A recommended merging step was tested on both 67 and 65 modules, respectively, and we observed that *HSD17B3* and *CYP11A1* were always in distinct modules.

These two WGCNA analyses performed in parallel allowed us to identify a consensus group of 335 genes including *HSD17B3* and a second group of 469 genes including *CYP11A1*. From the 18 genes presented in Figure 1, only *HSD17B3* is present in the cluster of 335 genes while 12 genes (*HSD11B2*, *CYB5R3*, *SRD5A1*, *CYB5A*, *CYP17A1*, *HSD17B4*, *LHCGR*, *STAR*, *SULT2A1*, *AKR1C2* and *DHRS4*) are included in the *CYP11A1* cluster (WGCNA-CYP11A1). A functional enrichment analysis underlined that the WGCNA-CYP11A1 group is effectively mainly related to steroid synthesis while the WGCNA-HSD17B3 is mainly related to gene transcription regulation and cell signaling. To complement this analysis, signatures of possible regulation of these genes by transcription factors (TF) were searched in upstream sequences of considered genes. We used the knowledge about the human orthologous corresponding gene sequences and transcription factors (TF) to calculate a relative enrichment score [35]. A higher relative enrichment was observed in the WGCNA-CYP11A1 than in the WGCNA-HSD17B3 for STAT1 (13.1, *P*: 4.1 × 10^−8^; 9.1, *P*: 1.3 × 10^−3^) and AR (25.1, *P*: 8.2 × 10^−10^; 16.7, *P*: 9.4 × 10^−4^). These analyses reported also a significant relative enrichment for NR5A1 (previously named SF-1 for Steroidogenic Factor 1) for genes from WGCNA-CYP11A1 (59.0, *P*: 1.8 × 10^−6^).

### 3.3. Characterization of a Correlation Group Around CYP11A1

**Figure 3:**
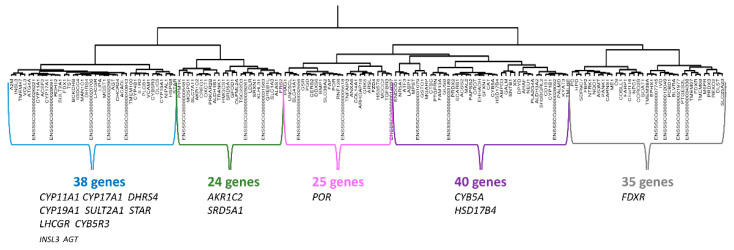
Presentation of CYP11A1-cluster-P162. An HCA was built with data (mRNAseq-P) observed for the 469 genes included in WGCNA-CYP11A1. The analysis of this clustering allowed the characterization of a group of 162 genes linked closely to *CYP11A1* (*C*YP11A1-cluster-P162). For the composition of sub-clusters, we have prioritized genes present in the group around *CYP11A1* characterized by RT-qPCR (see Figure 1).

A hierarchical clustering of 469 genes from the WGCNA-CYP11A1 group was performed on the mRNAseq-P dataset. When we chose to retain the top 100 genes with the largest variance, we observed that the sub-cluster defined by the strongest correlation links contained *SULT2A1*, *CYP11A1*, *CYP17A1*, *STAR*, *CYP19A* and *DHRS4* (see Supplementary Figure S3). In the second part of the tree, no gene previously cited in this study was identified. Consequently, to define a co-expression network with *CYP11A1* as hub gene, we performed an HCA with all 469 genes (Supplementary Figure S4) and we selected the subtree containing *CYP11A1* and other subtrees associated with it by strong correlation links (Figure 3). With this approach, we were able to identify a core group of 38 genes around *CYP11A1*. This group is itself inserted in a correlation cluster comprising 162 genes (CYP11A1-cluster-P162, Supplementary Tables S3 and S4). The other two clusters containing 87 and 220 genes can be excluded from the CYP11A1-group because they were linked to it by weak correlations (Supplementary Figure S4).

Thereafter, we selected all genes whose expression was strongly correlated to *CYP11A1* in the mRNAseq-P data set (r > 0.8, *p* < 0.000067). A list of 68 genes was established (CYP11A1/list-P68, see Supplementary Table S5). Among the 38 genes from the sub-cluster of CYP11A1-cluster-P162 containing *CYP11A1* (indicated in blue on the Figure 3), 28 genes are present in both lists. In the CYP11A1/list-P68, we found 2/24, 4/25, 17/40, and 8/35 genes from the sub-cluster indicated in green, pink, purple and grey, respectively. Only nine genes were not present in the CYP11A1-cluster-P162. In other words, 59 genes of the 68 of the CYP11A1/list-P68 are present in the CYP11A1-cluster-P162. All information was concordant to conclude that the CYP11A1-cluster-P162 correctly described the correlation group around *CYP11A1* in the mRNAseq-P dataset.

**Figure 4:**
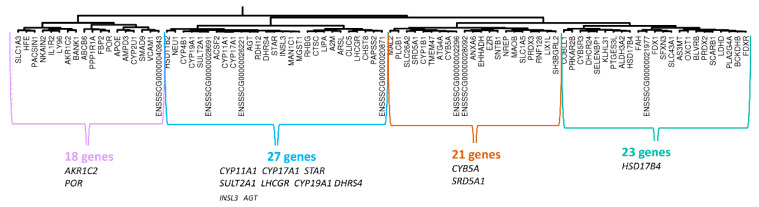
Presentation of CYP11A1-cluster-PLW89. An HCA was built with data (mRNAseq-PLW) observed for the 469 genes included in the correlation module previously defined by WGCNA analysis around *CYP11A1*. The analysis of this clustering allowed the selection of a group of 307 genes linked by correlations. In this figure, only the sub-cluster (89 genes) including *CYP11A1* is shown. For the composition of sub-clusters, we have prioritized genes present in the group around *CYP11A1* characterized by RT-qPCR (see Figure 1).

To define a co-expression network with *CYP11A1* as the hub gene with mRNAseq-PLW data, a new HCA was performed with 469 genes of WGCNA-CYP11A1. We selected the subtree containing *CYP11A1* and others subtrees associated with it by strong correlation links (Supplementary Figure S5). With this approach, we identified a core group of 27 genes whose expression is strongly correlated to *CYP11A1*. This group is itself inserted in a module of 89 genes (Figure 4) which is itself inserted in a module of 379 genes. The other two clusters with 23 and 67 genes can be excluded from the correlated group. Therefore, two provisional clusters were identified, the first CYP11A1-cluster-PLW89 was limited to 89 genes, and the second CYP11A1-cluster-PLW379 contained 379 genes (see Supplementary Tables S3 and S4).

We selected all genes whose expression was strongly correlated to *CYP11A1* in the mRNAseq-PLW dataset (r > 0.8, *p* < 0.000039). A list of 105 genes (CYP11A1/list-PLW105, see Supplementary Table S5) was established, and 67 were detected in the CYP11A1-cluster-PLW89. All the 27 genes from the sub-cluster of CYP11A1-cluster-PLW89 containing *CYP11A1* (indicated in blue on the Figure 4) were found in this list. For the other sub-clusters: 8/18, 15/21 and 17/23 genes from the sub-cluster indicated in purple, brown, and green, respectively were found in list-PLW105. This list contained still 38 genes exclusively in CYP11A1-cluster-PLW379. Considering that these analyses did not allow for the exclusion of new sub-clusters, we proposed to keep the CYP11A1-cluster-PLW379 to describe the correlation group around *CYP11A1* in mRNAseq-PLW.

**Figure 5:**
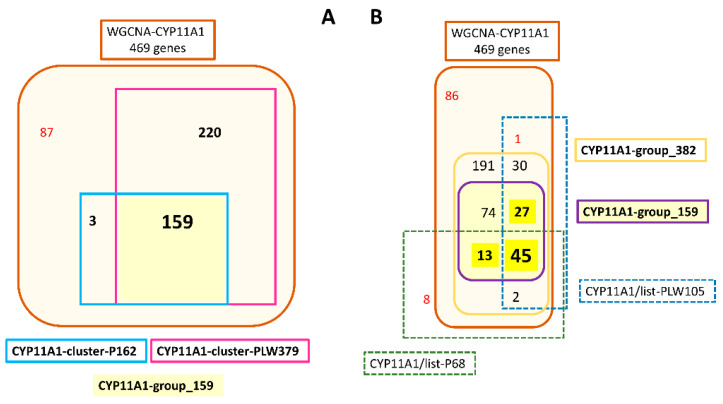
Analysis of genes likely to belong to CYP11A1-group. (**A**) The HCA analyses of HCAs led to defining two clusters around *CYP11A1*: CYP11A1-cluster-P162 and CYP11A1-cluster-PLW379 from WGCNA-CYP11A1. The comparison of the genes’ content of CYP11A1-cluster-P162 and CYP11A1-cluster-PLW379 allowed us to define CYP11A1-group_159 and CYP11A1-group_382 including 159 and 382 genes, respectively. (**B**) The comparison of genes’ contents of CYP11A1-group_159 and CYP11A1-group_382 and the two lists of genes highly correlated to *CYP11A1* (CYP11A1/list-P68 and CYP11A1/list-PLW105) led to characterizing of 45 genes constituting the core of CYP11A1-group. We suggest considering only the 45 + (13 + 27) genes as the correlation group around *CYP11A1.*

**Figure 6:**
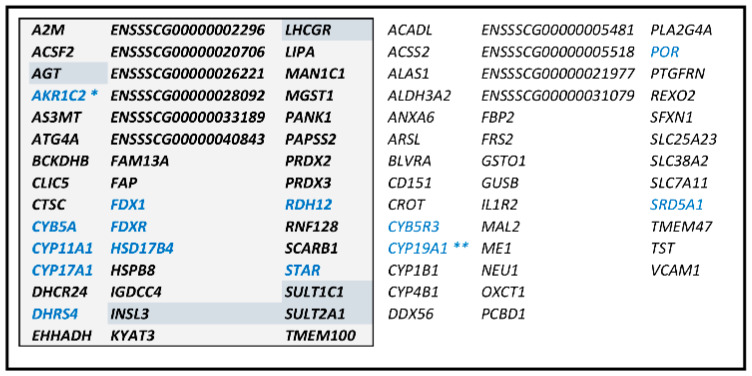
Presentation of CYP11A1-group (85 genes). The 45 genes presented in the shaded box constitute the core group. Their expression are highly correlated to that of *CYP11A1* in both datasets (mRNAseq-P and -PLW). The other 40 genes are strongly correlated to *CYP11A1* in only one dataset. All genes cited in Appendix A are indicated in blue. Genes that are indicated with a grey-blue background are genes cited in this study. * In mRNAseq analysis, the RSEM pipeline attributed to this gene counts of all AKR1C genes that have different known specificities. Nevertheless, RT-qPCR results (Figure 1) showed that the gene involved in testicular steroid synthesis is *AKR1C4*. ** In mRNAseq analysis, the RSEM counts attributed to this gene include counts of the three *CYP19A* genes.

CYP11A1-cluster-P162 and -PLW379 shared 159 genes and could constitute a large group of 382 genes (Figure 5A; Supplementary Table S5). The core of CYP11A1-group could be constituted by at least 44 genes with their expression highly correlated to that of *CYP11A1* (Figure 5B; Supplementary Table S5). Forty genes (13 + 27) were identified in both clusters and were present in CYP11A1/list-P68 or in CYP11A1/list-PLW105 (Figure 5B). Considering the cross-references with lists of genes whose expression is strongly correlated to *CYP11A1* (Figure 5B), we considered these 45 + 40 genes as a correlation group around *CYP11A1* (Figure 6). Two comprehensive tables including all genes concerned by the Figure 5B are proposed in Supplementary Tables S4 and S5.

### 3.4. Analyses of Correlation Group around HSD17B3

**Figure 7:**
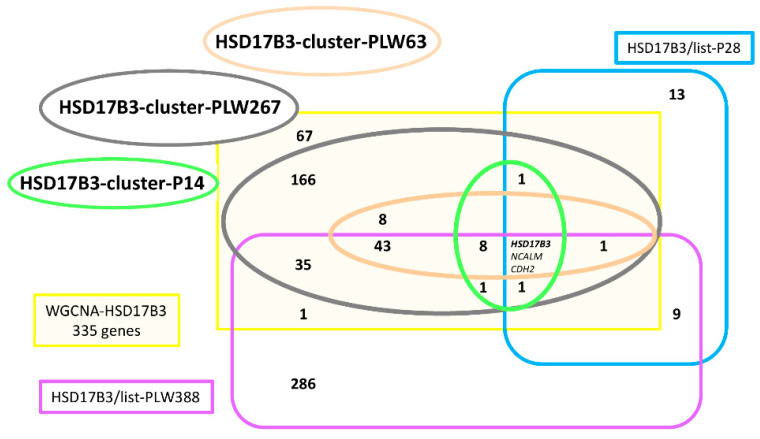
Characterization of the correlation group around HSD17B3. The analysis of HCAs led to defining HSD17B3-cluster-P14, HSD17B3-cluster-PLW63 and HSD17B3-cluster-PLW267 from WGCNA-HSD17B3. The list of genes strongly correlated to *HSD17B3* in each mRNA-seq was established: HSD17B3/list-PLW388 and HSD17B3/list-P28. The comparison between these five elements was not favorable to the characterization of a large correlation group around *HSD13B3.*

To define a co-expression network with *HSD17B3* as hub gene, an HCA on the mRNAseq-P dataset was performed with 335 genes identified (WGCNA-HSD17B3). This clustering (Supplementary Figure S6) did not highlight any group of clustered genes by strong correlation links. Nevertheless, we selected the part of the cluster (14 genes) containing *HSD17B3* (Supplementary Figure S7A). Among the 68 genes included in the list of genes strongly correlated to *HSD17B3* in this set of mRNAseq-P, only five appeared in the cluster retained (HSD17B3-cluster-P14) and 22 were not included in the WGCNA-HSD17B3. The analysis of the HCA performed with the 335 genes on the mRNAseq-PLW (Supplementary Figure S8) allowed to exclude 68 genes. The core group around *HSD17B3* seemed to consist of nine genes whose expression seem strongly correlated to *HSD17B3*. This group was itself inserted in a correlation module comprising 63 genes (Supplementary Figure S7B, Supplementary Tables S6 and S7), which is itself inserted in HSD17B3-cluster-PLW267. When we identified all genes, whose expression was strongly correlated to *HSD17B3* a list of 388 genes was established (HSD17B3/list-PLW388, Supplementary Tables S6 and S7). On this list, we found 54 genes from the HSD17B3-cluster-PLW63, but also 36 (+54) from HSD17B3-cluster-PLW267 (Figure 7). We have no argument to remove the 204 additional genes from HSD17B3-cluster-PLW267.

HSD17B3-cluster-P14 shared its 14 genes with HSD17B3-cluster-PLW267 (Figure 7) (Supplementary Table S7). The core of HSD17B3-group is composed of three genes: *HSD17B3, NCALM* and *CDH2*. If the same criteria that were used to define the CYP11A1-group are used here, the HSD17B3-group is limited to 14 genes. The HSD17B3/list-P28 and HSD17B3/list-PLW388 contained a very large proportion of genes not highlighted by WGCNA analysis, which was not the case for the CYP11A1/list-P68 and CYP11A1/list-PLW105.

When we used a new set of RNA-seq (six total-RNA-seq from animals of the same population) to identify all genes whose expression was strongly correlated to *HSD17B3* or to *CYP11A1*, we obtained very divergent results. The analysis revealed 307 protein-coding genes with their expression positively correlated (r > 0.91, *p* < 0.01) to *HSD17B3* (HSD17B3/list-PT307) and only 11 genes correlated to *CYP11A1* (Supplementary Table S7). Among the 307 genes present in HSD17B3/list-PT307, 210 were never highlighted in this study. For HSD17B3/list-P28 and HSD17B3/list-PLW388 the respective scores were now 10/28 and 223/388. Taking into account the weak overlaps between these analyses, *HSD17B3* did not appear as a hub gene in a possible correlation network.

### 3.5. Analysis of Possible Interactions of the Genes of CYP11A1-Group by Transcriptions Factors

**Figure 8:**
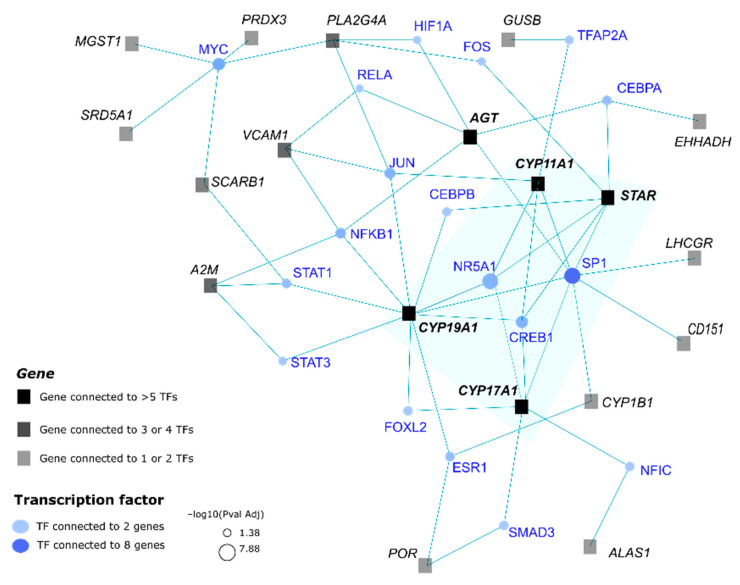
Possible regulation network of genes from the CYP11A1-group. Transcription factors (in blue) that may contribute to the regulation of genes (in black) from the CYP11A1-group were identified using human orthologous genes. The core (highlighted in a blue-green background) of this network included four genes and three TFs.

Potential upstream regulators of the CYP11A1-group were searched using the knowledge about the corresponding gene sequences and TF [35]. From the 85 genes (Figure 8), 82 orthologous human genes were selected for this analysis. The main network identified by this analysis integrated 19 genes having multiple interactions with 18 TFs. The main TF hubs were SP1 (Specificity Protein 1) and NR5A1, which were connected to eight and five genes of the CYP11A1-group respectively. The main hub genes were *CYP19A1*, *STAR*, *CYP17A1*, AGT and *CYP11A1*, which were connected to nine, six, six, five, and five TFs respectively. The core of this network seemed to be constituted of four genes (*CYP19A1*, *STAR*, *CYP11A1* and *CYP17A1*) and three TFs (SP1, NR5A1 and CREB1) (Figure 8). When we included *HSD17B3*, *NCALM* and *CDH2* in the input group of genes, no modification was observed.

### 3.6. GNRHR2—Preliminary Results on Mature Testis

A low transcriptional activity of *GNRHR2* was detected in the 37-mRNA-seq dataset but higher than the transcriptional activity of *AR* or *NR5A1.* In mRNAseq, it did not appear to be correlated with other genes previously highlighted. When we consider the three total-RNA-seq produced by EMBL from two-year-old boar testes, we cannot perform any correlation analysis, but we can examine the transcriptional activities of particular genes in these animals. While the transcriptional activity levels of *STAR*, *CYP11A1*, *CYP17A1*, *INSL3*, *CYP19A1* were similar for the EMBL-pig5 and -pig6 animals, the EMBL-pig4 animal was characterized by significantly lower transcriptional activities for the five genes. For *GNRHR2* activity, it was the opposite since the EMBL-pig4 animal had a significantly higher level. When comparing the transcriptional activity of *HSD17B3*, a similar level was observed in EMBL-pig4 and -pig6 whereas EMBL-pig5 was clearly higher.

## 4. Discussion and Conclusions

Analyses of correlations between transcriptional activities, measured by RT-qPCR, in testes of pubescent pigs allowed the identification of a group of 12 relevant genes. In this group, many genes are coding for key enzymes (*AKR1C4*, *CYP11A1*, *CYP17A1*, *CYP19A*, *HSD17B4*, *STAR*, etc.) of the steroid biosynthesis pathways (see Appendix A). Ten of these genes define a core group of co-expressed genes around *CYP11A1* while the two last genes, *HSD17B3* and *HSD3B1*, known to be important for the control of steroid synthesis, did not belong to this group. In this group of ten genes, most of them are involved only in steroid biosynthesis, whereas the enzyme encoded by *HSD3B1* has wider implications [5]. This difference probably explains the absence of the latter gene in the group of the ten correlated genes. However, in testes, the enzyme coded by *HSD17B3* is involved in a unique reaction: The transformation of androstenedione in testosterone (see Appendix A) [16]. For all animals selected in this study, levels of testosterone and 17β-estradiol (Supplementary Table S1) were much lower than those observed in mature animals (unpublished data). In this study, pigs were at an early stage of puberty. Using RT-qPCR, we observed a high (significant) correlation between the transcriptional activities of seven genes with the plasmatic level of 17-β-estradiol. In contrast, no significant correlation was observed between the testosterone plasmatic levels and gene expression. Plasmatic level of testosterone is known to be subject to variations: hours of the day, stress of the animal, etc. [36]. This may explain the absence of significant correlation with gene expression. The testicular production of testosterone must be able to respond to physiological demands. Besides, the results presented here are corroborated by the fact that 17-β-estradiol is a much better indicator of the pubertal stage of development than testosterone [37].

A hierarchical clustering revealed the important role in the testicular transcriptome of a group of genes containing both *CYP11A1* and *CYP17A1*. To define this group, a complete analysis of all correlations between the levels of transcripts was performed. WGCNA [29] was chosen because of its potential to unravel the gene regulatory architecture of complex traits [38,39]. This tool proposes to cluster genes into modules based on their co-expression across a set of samples. The two batches of mRNA-seq were analyzed in parallel to characterize modules of co-expression genes containing either *CYP11A1* or *HSD17B3*. In the WGCNA-CYP11A1 group, except *HSD3B1,* we found all genes previously described as Leydig cell-specific genes (*LHCGR*, *SCARB1*, *STAR*, *CYP11A1*, *CYP17A*1, and *INSL3*) [40,41]. *FSHR*, *DHH* and *SOX9,* previously described in mice as Sertoli cell-specific genes [42], were considered in the analysis but were not retained in both groups. In the WGCNA-HSD17B3 group, no genes considered as informative genes to analyze Sertoli cells transcriptome [43] were found. As the transcription factor STAT1 (signal transducer and activator of transcription 1) was proposed as potential master regulator driving the Sertoli cells transcriptional program [44], signatures of TF were searched in upstream sequences of considered genes. These analyses did not result in the recognition of the WGNA-HSD17B3 module as being derived from the Sertoli cell transcriptome. None of the reference genes used in single cells RNAseq for the identification of other testicular cells were found in both WGCNA modules [45]. After analyzing the composition of the WGCNA-CYP11A1 module, we suggest that this module belongs to the transcriptome of Leydig cells, but we have no suggestion for the WGCNA-HSD17B3 module. The consensus parts of these two modules were further analyzed in order to obtain two networks with *CYP11A1* and *HSD17B3* as hub genes. The WGCNA, HCA and direct correlations analyses allowed to define a very strong correlated group around *CYP11A1* and containing most of the key genes involved in testicular steroidogenesis. The existence of this group withstood the analysis of two batches of 18 and 19 mRNA-seq. The core of the CYP11A1-group is composed of 45 genes and this group includes more than 85 genes (Figure 6). *HSD17B3* appears completely excluded from the CYP11A1-group, whatever the analysis. WGCNA analyses also allow the characterization of a putative HSD17B3-group. Nevertheless, the HSD17B3-group did not appear to be as strongly correlated as the CYP11A1-group. In this CYP11A1-group, we found all genes, with the notable exception of *HSD3B1*, involved in the biosynthesis of pregnenolone and androstenedione: *STAR*, *CYP11A1* (+*FDX1/FDXR*), *CYP17A1* (+*CYB5A/CYB5R3* + *POR*) ([5], see Appendix A). All genes previously described as Leydig cell-specific genes (*LHCGR*, *SCARB1*, *STAR*, *CYP11A1*, *CYP17A*1, and *INSL3*) [40,41] and identified in the WGNA-CYP11A1 were retained in the CYP11A1-group. *POR*, *FDX1/FDXR* and *CYB5A/CYB5R3* are accessory proteins involved in the activity of enzymes encoded by *CYP11A1* and *CYP17A1*. The cyb5 reductase could be encoded by *CYB5R1* as suggested by Squires et al. [1] but the genes *CYB5R1* and *CYB5R3* were correctly considered in the present study and only *CYB5R3* appeared in the CYP11A1-group (in all the configurations explored). The CYP11A1-group contained also a gene encoding a 5α-reductase, *SRD5A1*. Its presence in this group confirms its involvement in the testicular production of 5α-reduced steroids (epiandrosterone, androstanediol, and androstenone) [1,18]. Among the core CYP11A1-group, we found two genes encoding for sulfotransferases. Steroid sulfation or sulfonation (or sulfoconjugation) make steroids more soluble and hence sulfated steroids by far outweigh unconjugated steroids in circulation [46]. In testes, the main gene involved in sulfonation of hydroxysteroids is *SULT2A1* [46,47]. A specific sulfotransferase is required for sulfation of estrogens because the hydroxyl group is an aromatic alcohol. In testicular transcriptomes analyzed in this study, the expression of *SULT1E1* was insignificant suggesting that that *SULT1E1* is not the gene coding a sulfotransferase as in human testes [48]. The gene ENSSSCG00000028691 referenced as *SULT1C1* in this study, due to its membership in the group of correlated genes, may be involved in the sulfonation of estrogens in testes. Besides, it may be speculated that epididymis and not testes are the major sites of sulfoconjugation of estrogens in pigs [49].

In this CYP11A1-group, we found all genes involved in the biosynthesis of estrogens (*STAR*, *CYP11A1*, *CYP17A1*, and *CYP19A*) except *HSD3B1*. This CYP11A1-group contained also *HSD17B4*, which codes the enzyme involved in the estrone-estradiol conversion [5]. The influence of this group of genes on the control of androstenone production is probably less marked. Indeed, we believe that the absence of *HSD3B1* in this group affects this control and the connections of *SRD5A1* to this group were probably weaker (RT-qPCR analyses: this study and [18]). In this list, we found genes able to code all enzymes required for testosterone synthesis (*STAR*, *CYP11A1*, *CYP17A1* and *AKR1C4*), except *HSD3B1*. The mRNAseq analysis did not allow us to identify which of the four AKR1C genes were present in the porcine genome, but RT-qPCR analyses showed that *AKR1C4* is indeed included in the CYP11A1-group. In humans, *AKR1C3* (names of human and porcine AKR1C genes are independent) supports testosterone production but only in adrenals [16]. The absence of *HSD17B3* in the CYP11A1-group suggests that testosterone is produced via two distinct synthesis pathways. The existence of two distinct testicular mechanisms to produce testosterone has been recently demonstrated in mice [50]. This double way is consistent with previous knowledge: The release of 17β-estradiol and testosterone have different age-related plasma patterns [51] and *HSD17B3* expression decreases along sexual maturation [14], whereas plasma testosterone levels are higher after 6 months of age than during post-natal period [52]. In the mRNAseq analyzed here, the normalized expression of *HSD17B3* and *AKR1C4* were similar. We identified the gene *LHCGR* in the CYP11A1-group and this gene encoding for LH receptor is known to be a Leydig cell-specific gene as many genes involved in steroidogenesis [40,41]. In this study, a correlation was found between *HSD17B3* and *FSHR* (RT-qPCR) but it was not confirmed by WGCNA analyses performed on mRNA-seq (yet the individual correlation between these two genes was significant) and not previously detected [18]. Moreover, the gene encoding for the FSH receptor (*FSHR*) is known to be a Sertoli cell-specific gene [42] and *HSD17B3* is known to be mainly expressed in Leydig cells [14]. We identified *AKR1C4* and *LHCGR* in the CYP11A1 group, while *HSD17B3* was excluded, so we propose to conclude that these two testosterone synthesis pathways have distinct regulations.

The CYP11A1 group did not contain only genes involved in steroid biosynthesis pathways since *AGT* and *INSL3* were found in this list. The gene *AGT* encodes for angiotensinogen, which is the precursor of angiotensin peptides. The renin-angiotensin system is known to be essential for ovulation in females but the testicular expression of the gene *AGT* was also described as affected by a selection for higher fertility in mice [53]. The gene *INSL3* encodes for a peptide (Insulin-like peptide 3) that is a member of the relaxin family of peptide hormones. In porcine testes, *INSL3* secreted by Leydig cells is released not only into the blood circulation but also into the interstitial compartment [54]. The level of plasmatic INSL3 directly depends on the number and differentiation state of Leydig cells but this peptide is suspected to act mainly at the testicular level in adults [55]. INSL3 is involved in the sperm production of adult boars [56]. Other genes were known to produce proteins having an autocrine/paracrine role in testes. GnRH2 and its receptor GnRHR2 were previously described as having an important autocrine/paracrine role in the testis biology [3,4,57,58]. In this study, only *GNRHR2* was studied and its expression did not appear to be correlated with other genes previously highlighted. Nevertheless, the very preliminary results presented here on testes of two-year-old boars suggest that the situation may change with the testicular maturation: *GNRHR2* could join the CYP11A1-group.

We explored the presence of transcription factors, which could be involved in the control of the expression of genes from the CYP11A1-group. The analysis of promoter sequences based on the human orthologous genes from the CYP11A1-group led to identify SP1 and NR5A1 (SF-1). The SP1 transcription factor is considered to be too widely produced in mammalian tissues to be informative in terms of regulatory specificity (reviewed by [59]). It was already showed in other species that SP1 and NR5A1 are involved in the regulation of transcriptional expression of *STAR* [60,61] and *CYP11A1* [60]. SP1 has been described as being able to regulate the *LHCGR* expression [62] and NR5A1 to participate to the regulation of the expression of *CYP19A1* [63]. The involvement of CREB1 in the control of steroidogenesis was already described in human ovaries [64]. Moreover, the composition of the core of the regulatory network identified in the present analysis is in agreement with literature data. Indeed, transcription factors NR5A1 and JUN cooperate to activate the *FDX1* promoter in mouse Leydig cells [65]. NR5A1 is also involved in the activation of the *INSL3* promoter in Leydig cells [66]. The gene *NR5A1* is included in the CYP11A1-group-159 (Figure 5) and the product of this gene is a receptor and a transcription factor. Present analysis seems very relevant, even though it was performed with the human sequence data. Finally, the only result obtained after using porcine sequences, was that the *GNRHR2* gene seems also regulated by NR5A1, CREB [57] and SP1 [67].

This study was performed using transcriptome from whole testes, with possibly a different proportion of cells types due to the physiological age of animals, and to the different genetic origins (two lines were included in our study). To overcome the problems due to this heterogeneity, we implemented an analytical approach based on the identification of common characteristics. Therefore, the heterogeneity between animals should only create “noise” that prevents the detection of certain relationships but does not lead to the identification of spurious relationships. Most of the genes of the CYP11A1-group showed a transcriptional activity with large variations between individuals (mRNA-seq). Even if the *SFXN1* or *NR5A1* genes are among the exceptions, it is likely that our approach to analyze mRNA-seq is not well adapted to the identification of relationships involving genes with little variation in their transcriptional activity (*HSD17B3*, *GNRHR2* or *AR*).

The present study was performed on boars of 20 to 26 weeks that are not fully sexually mature. Therefore, we cannot exclude that some features of the described testicular transcriptome are only transient. The very preliminary results presented here on testes from two-year-old boars suggest that the CYP11A1 group could still exist in mature testes. The integration of *GNRHR2* in this group is possible but this is probably not the case for *HSD17B3*. In order to study the nature of the involvement of *GNRH2/GNRHR2* in testicular hormone production, it will be necessary to study a cohort of mature animals.

This work allowed us to identify a group of genes including *CYP11A1* with highly correlated expression. Taking into account that many of these genes are involved in the testicular steroidogenesis or its regulation, we suggest that this group controls the major metabolic pathways leading to the synthesis of most of the testicular steroids. We emphasize that that the gene coding for the LH receptor is included in this group. We suggest that the presence of *HSD17B3* outside this group probably ensures that, at least, part of the testosterone production escapes this control. The porcine *AKR1C4*, which is in the CYP11A1-group, appears to be the best candidate gene for encoding a second active 17β-hydroxysteroid dehydrogenase in the porcine testis. This study provides new arguments to support the involvement of *SRD5A1*, *CYB5R3* and *SULT1C1* rather than *SRD5A2*, *CYB5R1* and *SULT1E1*, respectively, in metabolic pathways related to testicular steroid production in pig.

## Figures and Tables

**Figure A1:**
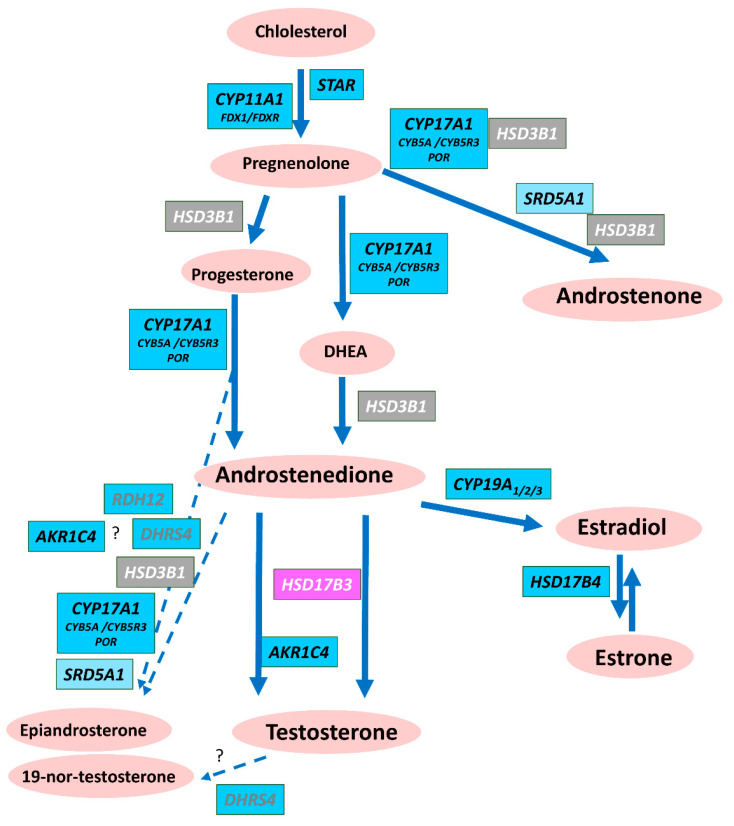
Landscape of genes involved in the pathway of sex steroids production in the porcine testis. The initial step to produce all sex steroids in the testes is the transformation of cholesterol in pregnenolone. In all mammals, the transformation of pregnenolone in androstenedione opens the common pathway to estrogens and androgens. Pregnenolone is also the starting product in the androstenone pathway. Epiandrosterone, 19-nor-testosterone, testosterone and androstenedione are androgens. 17β-estradiol and estrone are estrogens. Androstenone is a pheromone. The pathways of the biosynthesis of epiandrosterone and 19-nor-testosterone are not completely known.

## Data Availability

The data presented in this study are available on request from the corresponding author.

## Supplementary Materials

The following are available online at https://www.mdpi.com/article/10.3390/genes12040551/s1, Figures S1 and S2: WGCNA analysis; Figures S3–S5: WGCNA-CYP11A1; Figures S6–S8 WGC-NA-HSD17B3; Table S1: Animals; Table S2: primers; Tables S3–S5: WGCNA-CYP11A1; Tables S6 and S7: WGCNA-HSD17B3.

## Appendix A

The gene *STAR* encodes for the steroidogenic acute regulatory (StAR) enzyme.

The gene *CYP11A1* encodes for the cytochrome P450 side-chain cleavage enzyme (P450scc).

In the porcine species, the enzyme P450c17 encoded by the *CYP17A1* gene has three activities: 17α-hydroxylase, C17,20 lyase and andien-β synthase activity (pregnenolone ≫androstenone).

The gene *CYP19A_1/2/3_* encodes for the cytochrome P450 aromatase (P450aro).

The gene *HSD3B1* encodes for the 3β-hydroxysteroid dehydrogenase/Δ5-Δ4 isomerase (3β-HSD)

The gene *SRD5A1* encodes for a 5α-reductase responsible of the reduction of the Δ4–5 bond of 3-oxo (3-keto), Δ4,5 C19/C21 steroids.

All genes identified in the group of correlated genes involved in steroidogenesis are indicated in a blue box (*STAR*, *CYP11A1* (+*FDX1/FDXR*), *CYP17A1* (+*CYB5A/CYB5R3* and *POR*), *DHRS4*, *AKR1C4*, *CYP19A_1/2/3_*, *HSD17B4* and *RDH12*). *SRD5A1* that is slightly correlated to this group (RT-qPCR analyses) is indicated in a light blue box. *HSD3B1* and *HSD17B3*, for which correlations with this group were close to zero, are indicated in a grey and pink box, respectively.
