# Supplementary material for: Correlation Networks Provide New Insights into the Architecture of Testicular Steroid Pathways in Pigs
Source: Genes (Basel). 2021 Apr 9;12(4):551. doi: 10.3390/genes12040551 (PMC8069258; doi:10.3390/genes12040551)

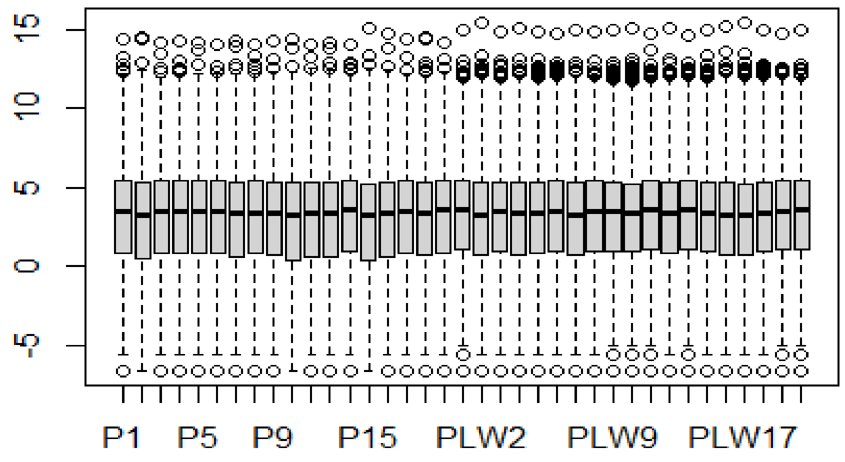

Figure S1A

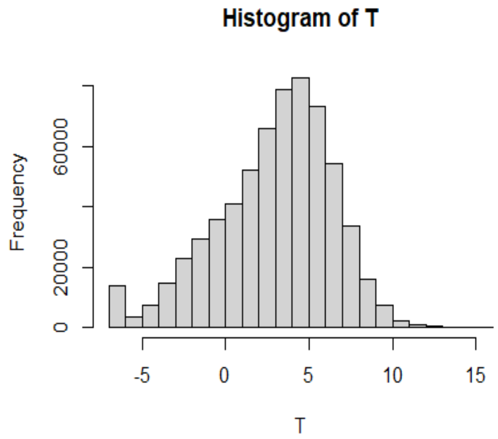

Figure S1B

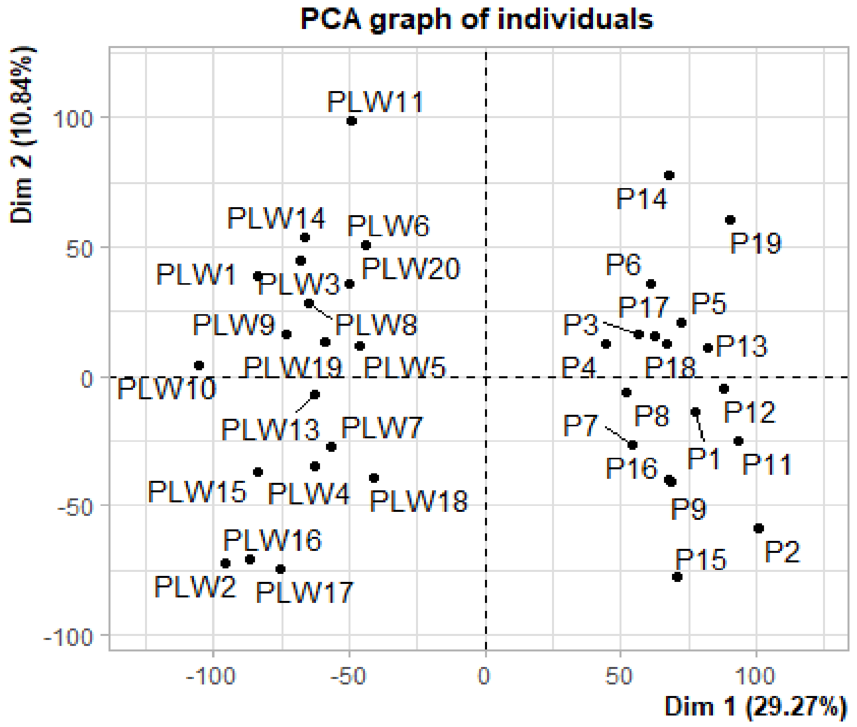

Figure S1C

Figure S1D

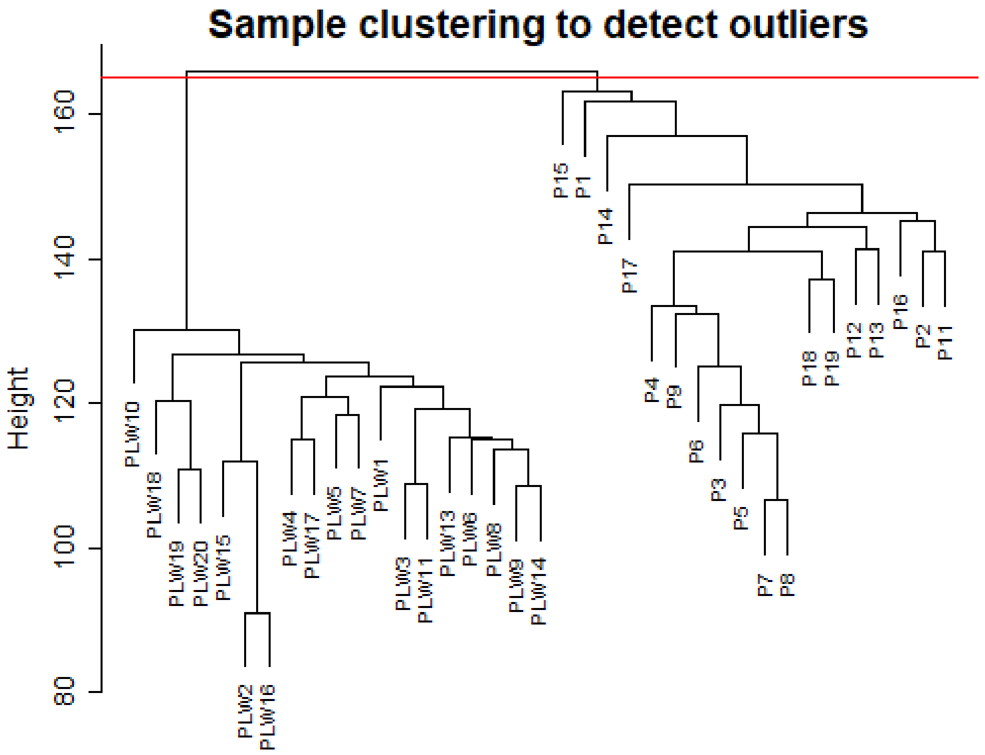

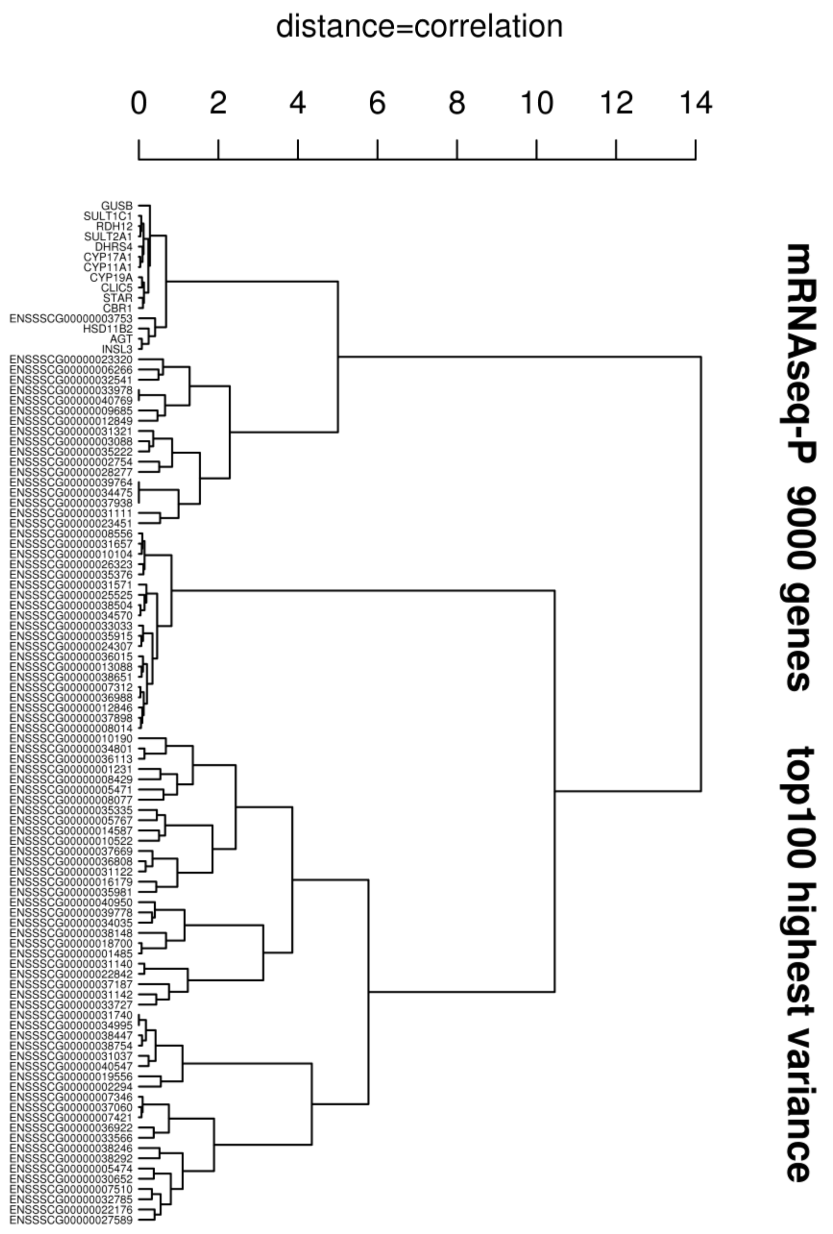

Figure S2A

Figure S2B

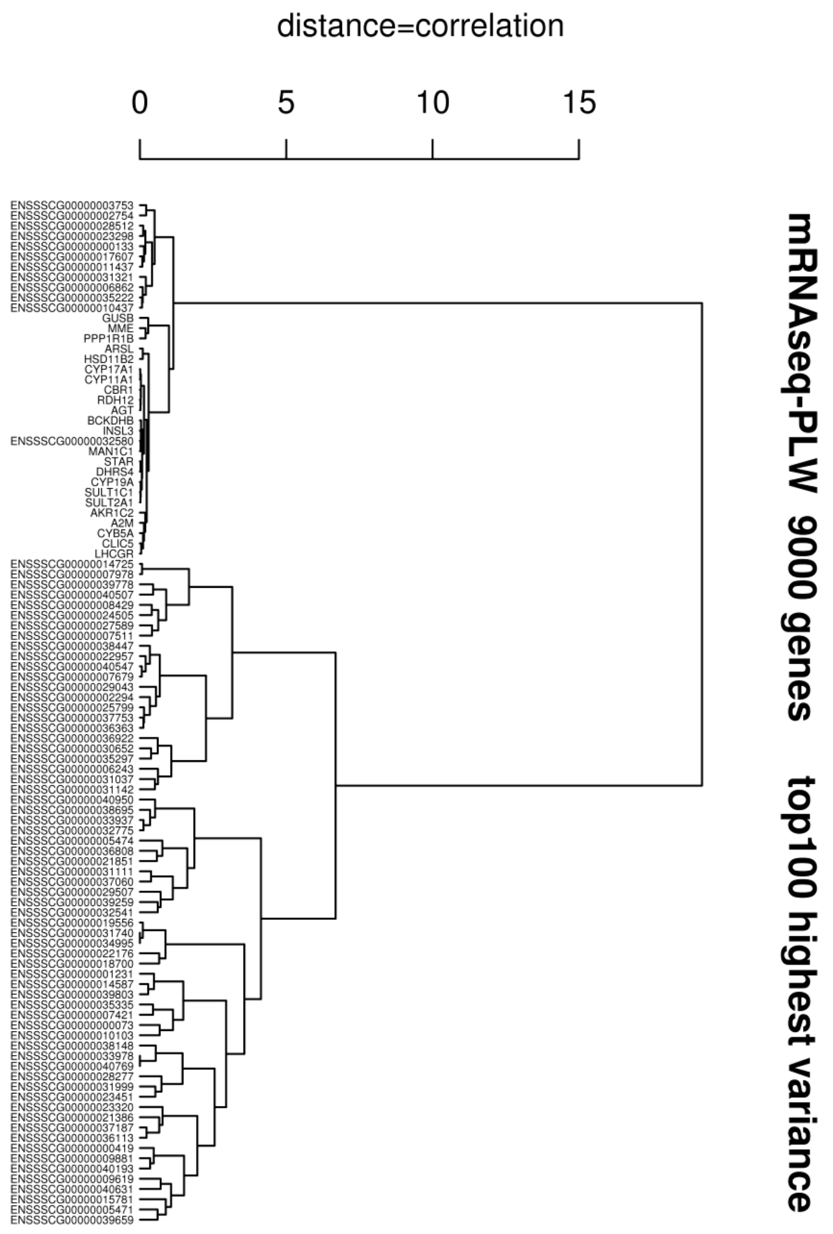

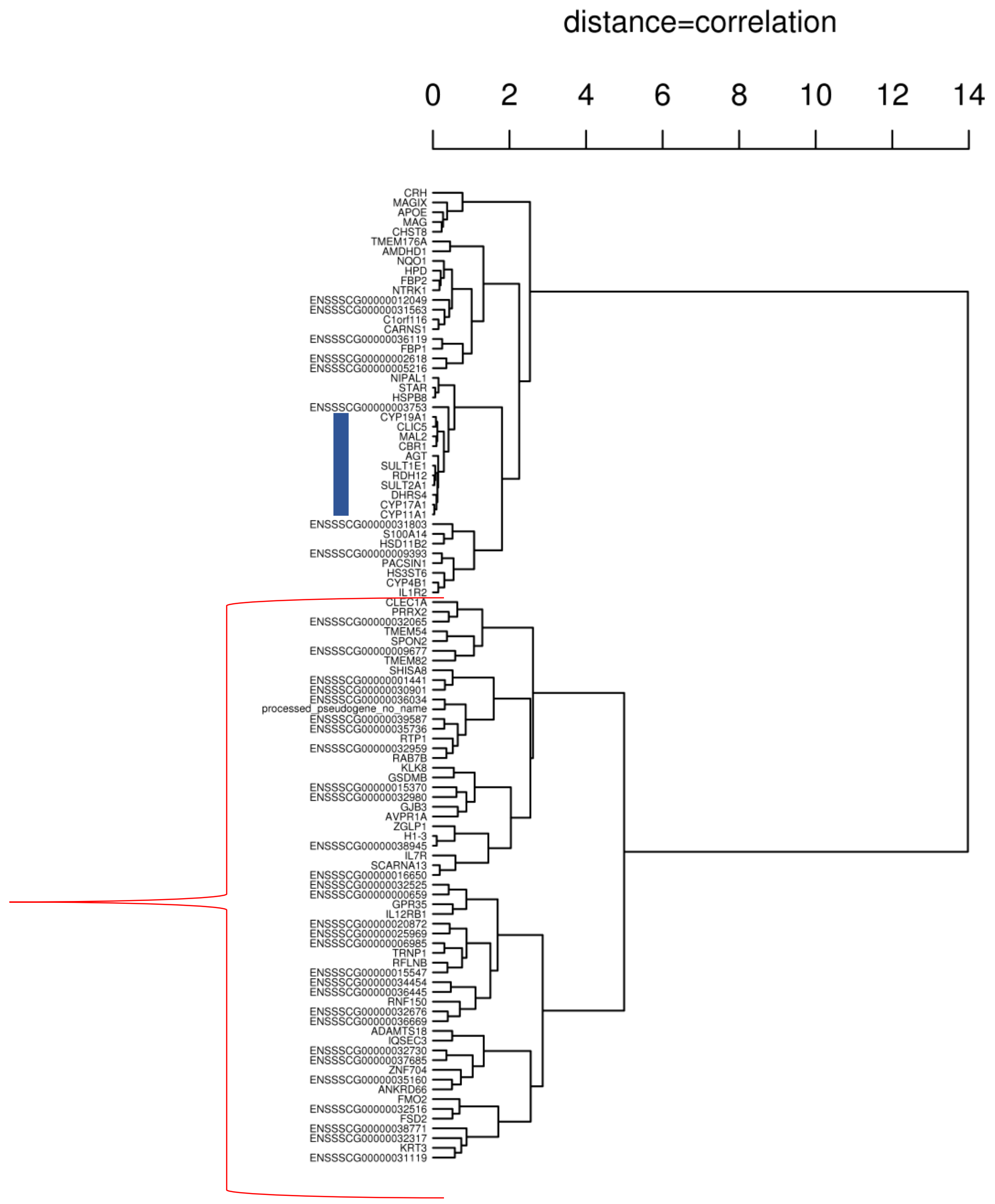

WGCNA-CYP11A1
469 genes
mRNAseq-P

Figure S3

The top 100 genes with the biggest variance were retained to perform the hierarchical clustering using correlation as distance (agglomeration: ward).

Figure S4 WGCNA−CYP11A1 (469 genes) mRNAseq−P

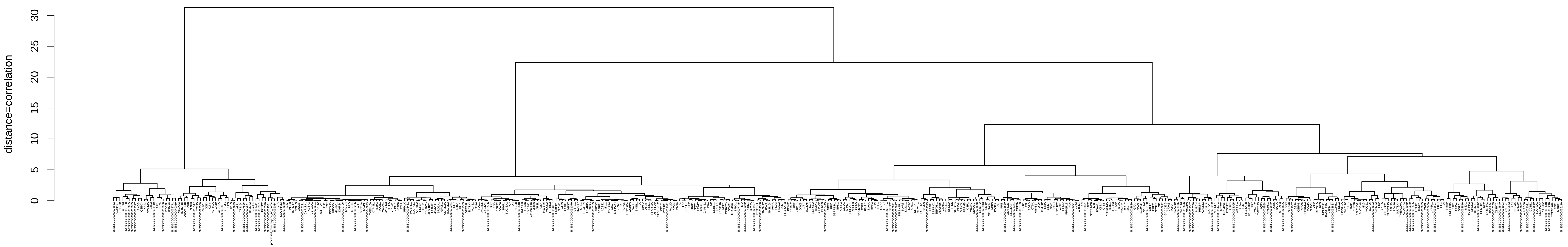

Figure S5 WGCNA−CYP11A1 (469 genes) mRNAseq−PLW

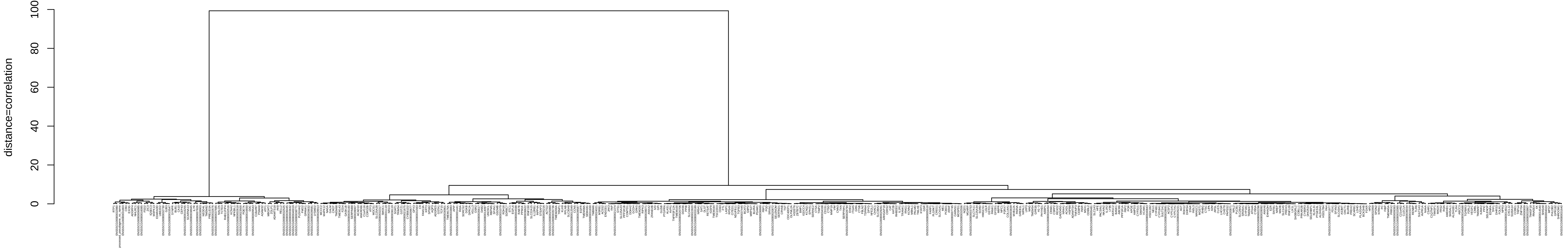

Figure S6 WGCNA−HSD17B3 (335 genes) mRNAseq−P

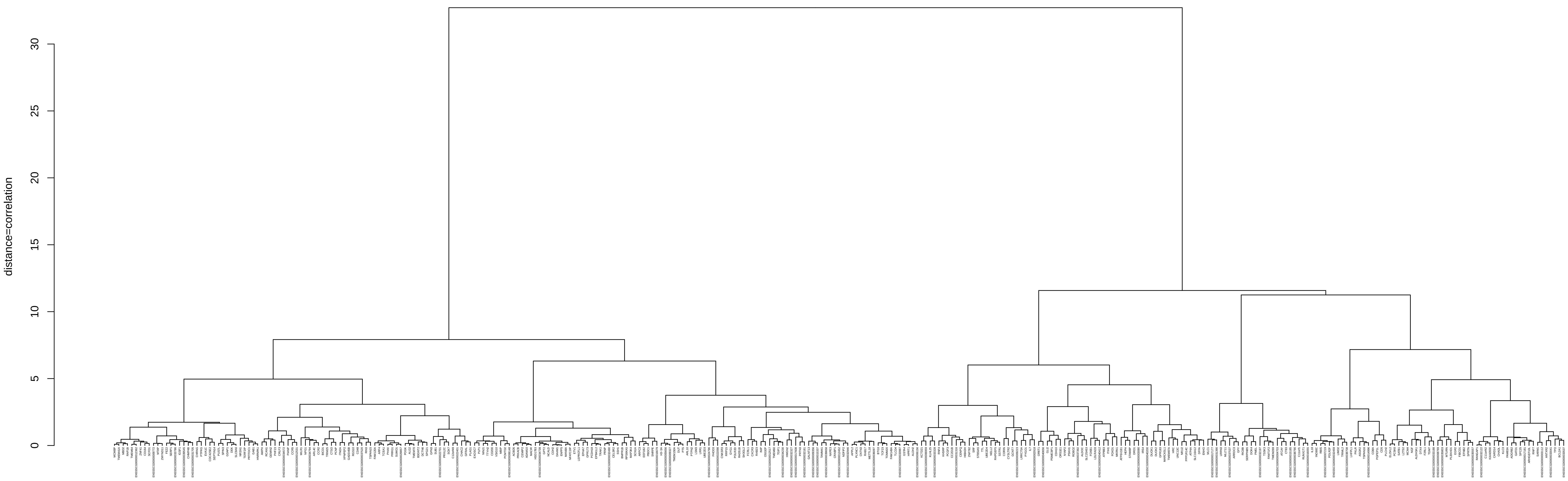

Figure S7A

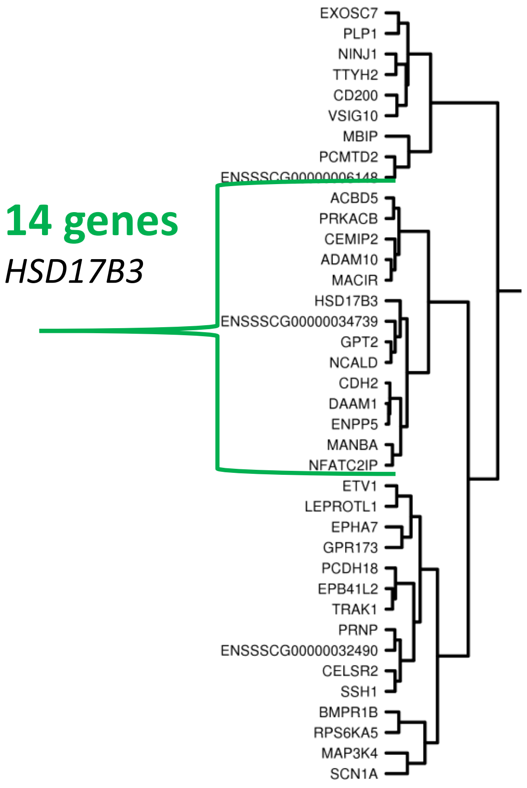

Figure S7B

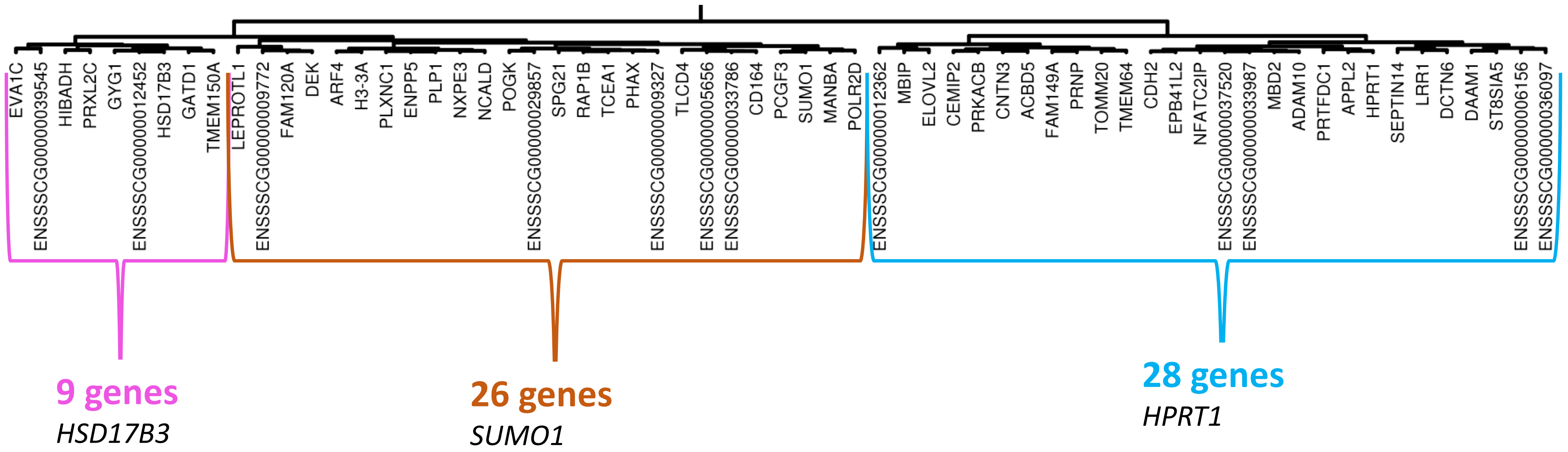

Figure S8 WGCNA−HSD17B3 (335 genes) mRNAseq−PLW

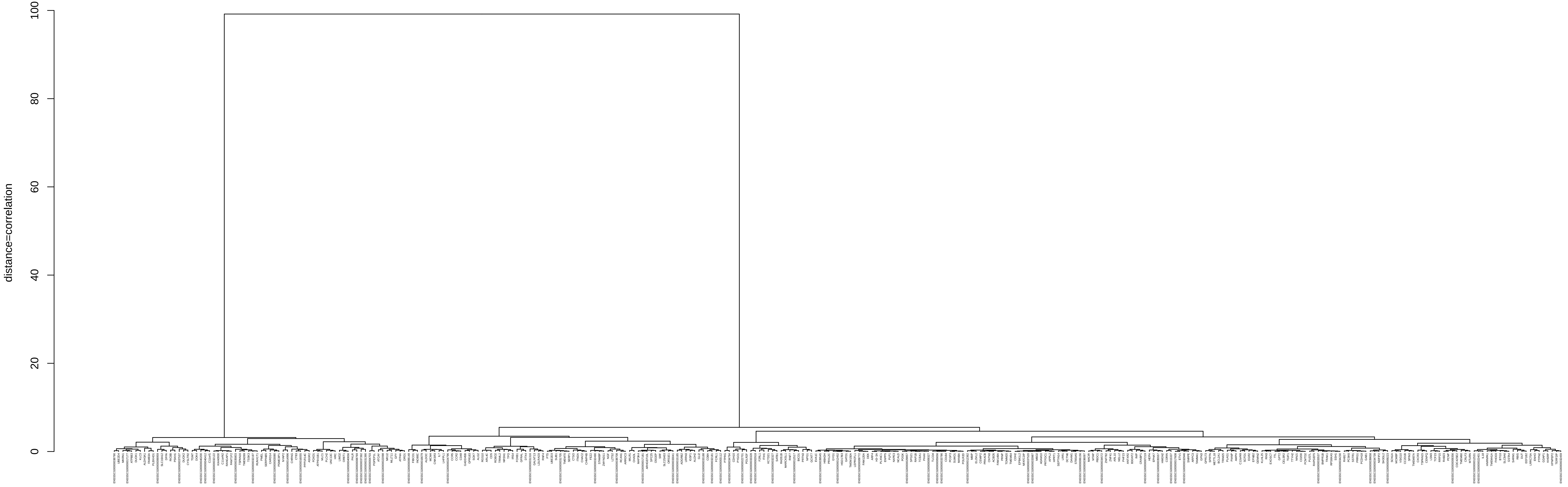

Supplement: Supplementary file 1 [file genes-12-00551-s001.zip › Supplementary-Figures.pdf]
